# Identification and Molecular Characterization of Telosma Mosaic Virus (TelMV) and East Asian Passiflora Virus (EAPV) from Patchouli in China

**DOI:** 10.3390/v16121837

**Published:** 2024-11-27

**Authors:** Asma Aziz, Na Li, Xiaoqing Wang, Linxi Wang, Yougen Wu, Muhammad Zeeshan Ul Haq, Zhaoji Dai, Hongguang Cui

**Affiliations:** 1 Key Laboratory of Green Prevention and Control of Tropical Plant Diseases and Pests (Ministry of Education), School of Tropical Agriculture and Forestry, Hainan University, Haikou 570228, China; 2 School of Breeding and Multiplication (Sanya Institute of Breeding and Multiplication), Hainan University, Sanya 572025, China

**Keywords:** *Pogostemon cablin* (patchouli), telosma mosaic virus (TelMV), symptoms, phylogenetic analysis, East Asian Passiflora Virus (EAPV)

## Abstract

Patchouli is a valuable medicinal herb and cash crop in China, but viral infections cause significant yield losses. This study identified six viruses in patchouli transcriptome data, including the first-ever detection of East Asian Passiflora Virus (EAPV) in patchouli. RT-PCR validated three viruses from diseased patchouli plants in Haikou, China: telosma tosaic virus (TelMV), broad bean wilt virus-2 (BBWV-2), and pogostemom alphacytorhabdovirus 1 (PogACRV1_Pog). The complete genomic sequence of TelMV from patchouli (TelMV) was determined, revealing a 9691-nucleotide RNA genome encoding a 3083-amino-acid polyprotein. Comparative analysis showed 77.66% to 81.01% nucleotide sequence identity with previously reported TelMV isolates. TelMV was also shown to be infectious in *Nicotiana benthamiana* through sap rub-inoculation. Additionally, a large portion of the EAPV genome was reconstructed from RNA-seq data, with coat protein analysis confirming its identity. This study presents the first complete TelMV genome in patchouli and the first detection of EAPV in the plant.

## 1. Introduction

*Pogostemon cablin* (patchouli) is from the *Lamiaceae* family and is highly valued for its essential oil. It is indigenous to South and Southeast Asia and widely cultivated in tropical regions, including China, Indonesia, and India [1,2,3]. In China, the plant is known as “Guanghuoxiang” [4] because of its medicinal properties, such as anti-inflammatory, antibacterial, antipyretic, anti-nausea, and antidepressant [5,6,7] properties. Patchouli is an herbaceous plant, and the oil-producing glands are on the plant’s leaves. Patchouli oil is a thick liquid that ranges from yellowish green to brown and has a solid and long-lasting scent. Patchouli oil is a robust fixative agent frequently used in perfumery, cosmetics, and pharmaceutical industries [8]. It ranks highly among the top-20 essential oil-producing plants traded globally and is included in the Chinese Pharmacopoeia due to its medicinal significance [9,10,11,12].

Patchouli cultivation is seriously threatened by various diseases caused by fungi, viruses, and nematodes. The annual worldwide production of patchouli herb for essential oil production is approximately 1200 tons, falling short of the 1600 tons demanded annually [13]. Viruses are a more severe hazard than other pathogens, resulting in substantial economic losses for the industry and farmers. Recent studies indicated that various viruses, particularly potyviruses and fabaviruses, are prevalent in patchouli cultivation, causing detrimental effects on plant health and yield [14]. Patchouli plants are documented to be infected by nine viruses [15], including patchouli mild mosaic virus (PatMMV), patchouli mosaic virus (PatMoV), patchouli yellow mosaic virus (PatYMV), telosma mosaic virus (TelMV), peanut stripe virus (PStV), tobacco necrosis virus (TNV), broad bean wilt virus-2 (BBWV-2), cymbidium mosaic virus (CymMV), and cucumber mosaic virus (CMV). Some of these viruses have been widely investigated. Many patchouli viruses remain undiscovered or only partially understood, making virus research crucial for developing effective management strategies.

TelMV is a positive-sense single-stranded RNA (+ssRNA) virus classified under the genus *Potyvirus* within the family *Potyviridae*. It poses a significant threat to the cultivation of patchouli, leading to mosaic symptoms that reduce leaf yield and decrease the oil content of the leaves. The viral genome consists of a large polyprotein cleaved into 10 mature proteins by the viral protease [16,17,18,19]. A key feature of potyviruses is the presence of a small open reading frame (ORF) known as Pretty Interesting *Potyviridae* ORF (PIPO), which is generated due to a slippage event during RNA polymerase replication [20,21,22,23]. TelMV has been previously detected in *Telosma cordata*, *Passiflora edulis*, and *Senna alata* in countries such as Vietnam [24], Thailand [25], and China, respectively [26,27]. The virus was first identified in patchouli in Indonesia [28], and, more recently, our lab confirmed its presence in patchouli in China by analyzing data from the NCBI patchouli transcriptome database [29]. While a partial genome sequence of TelMV from Indonesian patchouli was submitted in 2016, the complete genome sequence of a TelMV isolate from patchouli has yet to be published.

Identifying and analyzing the sequences of additional isolates worldwide is crucial for comprehending the virus’s molecular diversity and evolution. The study’s aims were focused on (1) the identification of TelMV and other viruses from patchouli plants in China using transcriptomic data from patchouli plants and (2) the dissection of the molecular features of TelMV patchouli isolate.

## 2. Materials and Methods

### 2.1. Identification of Viral Sequences from Patchouli Plant Transcriptomic Data Analysis

The transcriptome data of six patchouli plants from a previous investigation were analyzed to identify viral sequences [30]. In order to filter low-quality sequences and trim adapter sequences, Trimmomatic software version 0.39 was implemented [31]. Following preprocessing and quality control steps, the reads were mapped to the host genome sequence using Bowtie 2 to remove any host-related sequences. Unmapped reads, which included RNA from fungi, bacteria, and other non-host species, were assembled de novo using the Velvet software. The resulting contigs were subjected to taxonomic profiling using Prodigal-2.6.2 (https://github.com/hyattpd/prodigal/wiki) (accessed on 5 February 2024) to identify open reading frames (ORFs). BLAST analysis compared these ORFs with the NCBI non-redundant protein sequences (Nr) database. The viral sequences were additionally verified through BLASTn and BLASTx analysis. A candidate for a novel or known virus was defined as a sequence that shared 50% to 90% similarity with previously known viral sequences. In order to verify the position of the contigs to the reference genome, the full-length genome and fragmentary nucleotide sequences of each virus were aligned with the reference genome.

### 2.2. Identification of TelMV from Infected Patchouli Plants in the Field by RT-PCR

Two patchouli plants that exhibited the chlorosis and mosaic symptoms indicative of viral infection were selected from the experimental field cultivated in a greenhouse at 20–28 °C under natural sunlight for further investigation. Two pairs of primers were designed according to the alignment position of contigs with reference genome selected from the NCBI database in the BLASTx comparison function (Table S1). The primers were synthesized by Sanger company (Shenggong Bioengineering Co., Ltd., Shanghai, China). Total RNA from both plants was extracted following the protocol provided in the EasyPure Plant RNA kit (TransGen Biotech, Beijing, China), and cDNA was synthesized using a RevertAid First Strand cDNA Synthesis Kit (Thermo Fisher Scientific, Waltham, MA, USA). PCR amplification was performed using Thermo Scientific Phusion High-Fidelity DNA Polymerase (Thermo Fisher Scientific) in a total reaction volume of 50 μL. The reaction components included 10 μL of 5× Phusion HF Buffer, 1 μL of 10 mM dNTPs, 1 μL of forward primer, 1 μL of reverse primer, 1 μL of template DNA, 0.5 μL of Phusion DNA Polymerase, and nuclease-free water to adjust the final volume to 50 μL. The PCR program consisted of an initial denaturation at 98 °C for 30 s, followed by 35 cycles of denaturation at 98 °C for 10 s, annealing at 55 °C for 30 s, and extension at 72 °C for 15–30 s per kb. The reaction was completed with a final extension at 72 °C for 10 min. The amplified PCR products were analyzed using gel electrophoresis, with the presence of expected bands confirming a positive result for viral infection. The PCR products were cloned into a pTOPO-Blunt vector (Aidlab Biotechnologies, Beijing, China) and sequenced using Sanger sequencing for further analysis. The obtained sequences were compared with RNA-seq contigs to confirm the identity of the identified viruses.

### 2.3. Assembly of TelMV Whole Genome from Infected Patchouli Plant

To obtain the complete genome of telosma mosaic virus (TelMV) isolate patchouli, we designed 10 primer pairs based on viral sequences discovered in RNA-seq data (Table 1). These primers were designed in an overlapping manner to amplify the nearly entire genome via RT-PCR (excluding 5′ and 3′ untranslated regions (UTRs)). Total RNA was extracted from symptomatic field-collected leaves using the RNAprep Pure Plant Kit (Tiangen, Beijing, China). Complementary DNA (cDNA) synthesis was performed using the RevertAid First Strand cDNA Synthesis Kit (Thermo Fisher Scientific). PCR was performed using Thermo Scientific Phusion High-Fidelty DNA Polymerase (Thermo Fisher Scientific), as described in the above section. Amplicons were visualized using 1% agarose gels in TAE buffer, and bands of the expected size were excised and purified from the gel. The PCR products were cloned into the pTOPO-Blunt vector (Aidlab Biotechnologies) and sequenced in triplicate using Sanger sequencing (Sangon Biotech, Guangzhou, China). The 5′ and 3′ terminal cDNAs were obtained using the 5′ and 3′ RACE kits (Invitrogen; Thermo Fisher Scientific, Waltham, MA, USA). The entire genome was assembled in a contig by using Seqman Pro 7.1.0 (Lasergene, GATC Biotech) to align overlapping sequences.

### 2.4. Sequence Similarity and Phylogenetic Analysis

The putative cleavage site patterns in the TelMV polyprotein were identified using the online website (http://www.dpvweb.net/potycleavage/index.html) (accessed on 15 July 2024). We employed BLASTn against the nt/Nr databases to identify the TelMV from the patchouli isolate’s closest relatives. The reference genome sequences from other potyviruses and all the reported sequences of TelMV were obtained from the NCBI GenBank database. For the phylogenetic analysis of the EAPV genome, 26 representative sequences of EAPV strains AO and IB and outgroup from other potyviruses were downloaded from the NCBI database. ClustalW was employed to align the selected nucleotide sequences, which were subsequently employed to generate a neighbor-joining phylogenetic tree in MEGA 11 [32] with a p-distance nucleotide substitution model. The 1000 bootstrap replicates were implemented to evaluate the robustness of the inferred evolutionary relationships.

### 2.5. Pathogenicity Test of TelMV on Nicotiana benthamiana Plants

To check the infectivity of TelMV on the indicator plants, *N. benthamiana* (*n* = 40) were grown in a growth chamber with a light/dark photoperiod of 16/8 h and a temperature difference of 22/18 °C (day/night). To prepare the inoculum, the infected leaves were ground in a mortar and pestle in 10 mM phosphate buffer (pH 7.5). Two leaves of four-week-old *N. benthamiana* were gently dusted with carborundum powder. The sap was lightly rubbed on the leaves using the index finger. After 5 min, the plants were rinsed with tap water. The leaves of mock-inoculated control plants were gently dusted only with carborundum powder. At 30 days post-inoculation (dpi), RT-PCR was conducted using the primers (Table S2), designed based on RNA-seq contigs, to verify the active infection of 4 viruses (TelMV, EAPV, PogACRV1_Pog, and BBWV-2) in the inoculated *N. benthamiana* plants. Total RNA was extracted from leaves using the RNAprep Pure Plant Kit (Tiangen, Beijing, China). Complementary DNA (cDNA) synthesis was performed using the RevertAid First Strand cDNA Synthesis Kit (Thermo Fisher Scientific). PCR was performed as mentioned in Section 2.2 using Thermo Scientific Phusion High-Fidelty DNA Polymerase (Thermo Fisher Scientific). The PCR products were subjected to gel electrophoresis, and the detection of the expected bands indicated a positive result for viral infection.

## 3. Results

### 3.1. De Novo Assembly of Patchouli Transcriptome for Virus Identification

**Figure 1:**
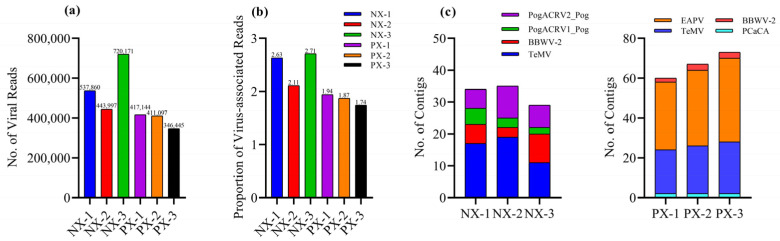
(**a**) Total no. of viral reads in each transcriptome. The viral reads were calculated by summing all viral reads present in each library. (**b**) The proportion of viral-associated reads in each library. (**c**) A stacked column chart displays the number of contigs assigned to different viruses in each transcriptome.

We analyzed six transcriptome datasets from two patchouli cultivars, Nanxiang (NX) and Paixiang (PX), each comprising three libraries (NX-1, NX-2, NX-3, PX-1, PX-2, and PX-3), available in the NCBI Read Archive under accession numbers (PRJNA660501 and PRJNA660544) to identify the viruses infecting patchouli. After trimming and preprocessing the data, 346,445 to 720,171 viral reads were obtained from the 6 libraries (Figure 1a). These reads were de novo assembled into contigs using Velvet software, resulting the variable number of viral contigs across the library (Figure 1c). The contigs were subjected to a BLASTx search against the Nr database (Table 2), identifying a nearly complete genome sequence of TelMV (excluding 5′ and 3′ UTR) and other patchouli viruses. These included *Potyviridae* (EAPV), *Secoviridae* (BBWV-2), and *Rhabdoviridae* (PCaCA, PogACRV1_Pog, and PogACRV2_Pog). This sequence mapping and BLAST analysis approach provided complementary datasets for each virus, as detailed in Table S3.

### 3.2. Identification of TelMV from Infected Patchouli Plants in the Field

**Figure 2:**
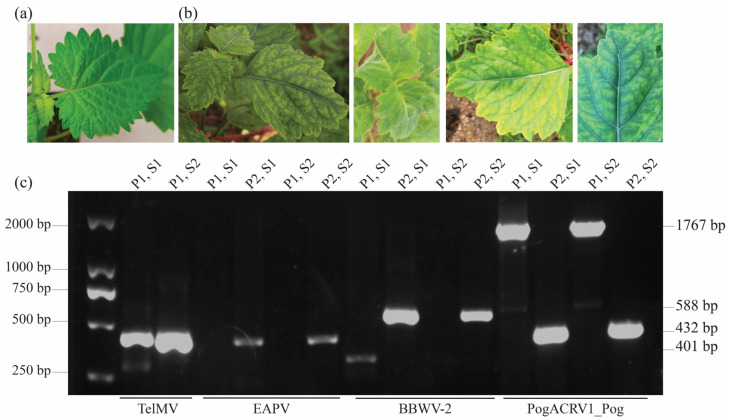
Identification of viruses from the infected patchouli plants. (**a**) Healthy plants showing dark green leaves. (**b**) Total RNA was extracted from the leaves of infected plants exhibiting crinkle and mosaic symptoms to be used for the RT-PCR test. (**c**) Agarose gel showing virus-specific DNA fragments amplified by RT-PCR assay using one pair of primers for TelMV and two pairs of primers to identify other viruses from two infected plants. P is for the primer pair, and S is for the sample.

Compared with healthy plants (Figure 2a), two patchouli plants exhibiting disease symptoms (Figure 2b) were selected from the field to identify TelMV. Total RNA was extracted from these symptomatic plants and used as a template for RT-PCR analysis. A single pair of primers, specific to TelMV, was employed to screen for the virus in the infected plants. Additionally, to accurately detect other viruses present in the samples, two pairs of primers were designed based on RNA-seq data. The RT-PCR results confirmed the presence of TelMV and two other viruses, producing amplicons of the expected size (Figure 2c). TelMV was detected in both plants, with an amplicon size of 430 bp. BBWV-2 was also positively identified in both plants, with a pair of primers, yielding an amplicon of 588 bp. The PogACRV1_Pog was detected using two pairs of primers, producing amplicons of 1767 bp and 432 bp, respectively, in both plants. However, the detection of EAPV was ambiguous. A faint band was observed on the gel with one pair of primers, suggesting a possible false-positive result, while no amplification occurred with the second pair of primers. For PogACRV1_Pog and PCaCA, no bands were amplified with either primer pair, confirming negative results for these viruses.

To further verify the identities of the detected viruses, the amplicons were subjected to Sanger sequencing. Sequence analysis revealed 100% identity in BLAST analysis and the contigs derived from the RNA-seq data, confirming the accuracy of the viral detection in the infected patchouli plants.

### 3.3. Obtaining the Complete Genome Sequence of TelMV Patchouli Isolate

The BLAST analysis identified 119 contigs, ranging from 200 bp to 1008 bp, related to the genomic sequences of TelMV. These short contigs were assembled into long contigs in Seqman Pro 7.1.0 (Lasergene, GATC Biotech), ranging from 320 bp to 4507 bp. The contigs were aligned with the reference genome to check their relative positions with the reference genome. In order to amplify the gaps between contigs, 10 segments were amplified by RT-PCR to get the whole genome sequence of the TelMV from patchouli. Two additional fragments, which correspond to 5′ and 3′ RACE results, were also recovered. The overlapping sequences of all these segments were combined to produce the entire genome sequence of the TelMV from patchouli. The complete genome of TelMV from patchouli plants is 9691 nt long, excluding the poly (A), which is within the range of genome lengths described for the Hanoi isolate but smaller than the genomes of passionfruit isolates. The TelMV from the patchouli genome contains a large open reading frame of 9248 nt that encodes a polyprotein of 3083 amino acids (aa). The virus’s 5′ untranslated region (UTR) is 189 nt in length and precedes the initiation codon. The 3′ UTR is 253 nt in length and is located downstream from the polyprotein, prior to the poly (A) tail. The assembled genome sequence was deposited at NCBI under accession no. PQ306486.

**Figure 3:**
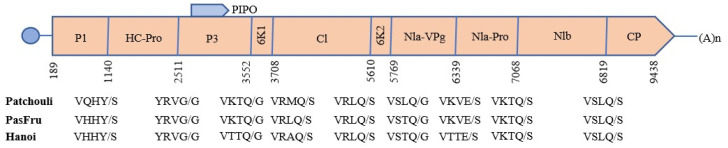
Genomic organization and cleavage-sites of TelMV patchouli genome. The circle is the Vgp (viral genome-linked protein), the large box is the long ORF (nucleotide 189-9438), small boxes show each mature protein resulting from proteolytic processing, and the small gray box represents the PIPO protein (nucleotide 2965-3184). An (n) represents poly A tail. Each protein’s protease cleavage site is indicated below for patchouli, passionfruit, and Hanoi isolates.

Like most viruses in the *Potyviridae* family, a Conserved Domain (CD) search revealed that the polyprotein of TelMV from patchouli exhibits a conserved organization in both the central and carboxy-terminal regions. The polyprotein is proteolytically cleaved into 10 mature proteins (P1, HC-Pro, P3, PIPO, 6K1, CI, 6K2, NIa-VPg, NIa-Pro, NIb, and CP) whose proteolytic cleavage sites (Y/S, G/G, Q/G, Q/S, Q/S, Q/G, E/S, Q/S, and Q/S) were in consensus with other TelMV isolates (Figure 3). The polyprotein was analyzed for conserved domain characteristics of potyviral proteins. The P1 conserved motif (^346^H-X8-E-X31-S^467^), representing a catalytic triad, was identified. The characteristics domain in HC-Pro (^2161^G-X-C-X31-L-X2-W-P-X36-H-V^2390^), which acts as its putative active site, the conserved motif (^1678^FRNK^1689^) involved in RNA silencing suppression activity, and (^1297^KLSC^1307^) and the (^2065^PTK^2073^), which function in the binding of HC-Pro to the virions for aphid transmission and systemic movement, were identified. The conserved motifs for helicase activity in CI (^3970^G-S-G-K-S-X3-P^3996^) and (^4228^D-E-X-H^4239^) were found. A motif in NIb (^8119^GDD^8127^) associated with replicase activity was also found. A conserved motif (^8641^DAG^8649^) was also located near the N-terminus of CP. All these motifs were identical to those of other TelMV isolates.

### 3.4. Characterization and Phylogenetic Analysis of TelMV

The NCBI databases BLASTn searches revealed that the CP nucleotide sequence was most similar to the different TelMV isolates, with similarity levels ranging from 83.93% to 88.99%. The CP nucleotide sequence shared 96.93% identity, with a query coverage of 51%, with the CP sequence of TelMV from the patchouli plant. These levels are higher than the threshold that is currently accepted for species demarcation in the *Potyviridae* family, which is between 76% and 77% identity for the CP nucleotide sequence. This indicates that the assembled genome sequence is from the same species. The complete genome of TelMV from patchouli shared 77.66–78.18% nucleotide sequence identities with previously reported TelMV isolates from passionfruit (MG944249, MK340754, MN316594, MK340755, and MT557572). Moreover, the patchouli isolate showed 81.88% similarity with the TelMV Hanoi isolate (NC009742), suggesting that the TelMV from the patchouli isolate was more closely related to the Hanoi isolate. The pairwise sequence comparison of all genome segments at nucleotides was 69.18–88.67% and 70.59–93.36 with PasFru and Hanoi isolates, respectively (Table 3).

**Figure 4:**
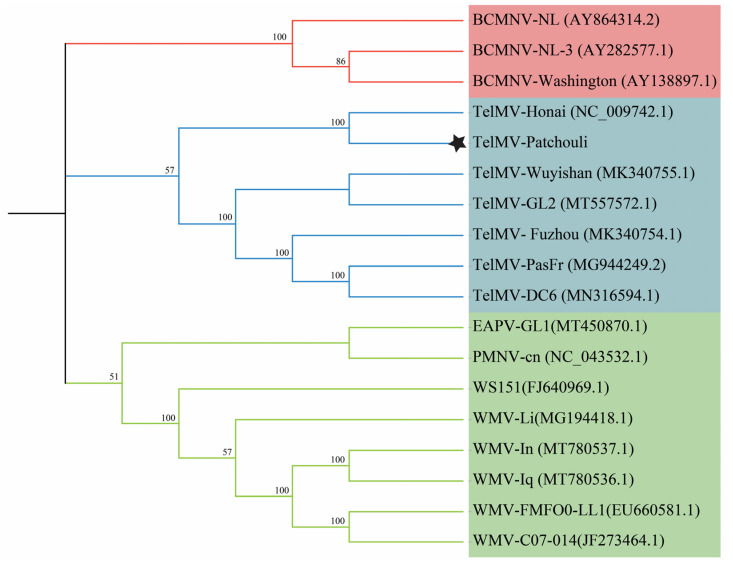
Phylogenetic analysis of TelMV from patchouli. The tree was constructed using the neighbor-joining algorithm, and the sequences were aligned in ClustalW using MEGA11. The p-distance was employed to calculate the evolutionary relation, and the numbers at the end of the branches represent the bootstrap values derived from 1000 replicates. Full names of viruses used in the phylogenetic tree (EAPV = East Asian Passiflora Virus, BCMNV = bean common mosaic necrosis virus. TelMV = telosma mosaic virus, PMNV = paris mosaic necrosis virus, and WMV = watermelon mosaic virus). Asterisk denotes the TelMV from the patchouli plant.

A neighbor-joining phylogenetic tree, comprising TelMV from patchouli complete genome sequence and TelMV genome sequences from other hosts, yielded a similar result. The evolutionary relationship was analyzed by selecting outgroups from the potyviruses, which contained sufficient homologous sites for the respective ingroup virus species, and ingroups were selected from the same species available on NCBI GenBank. The generated phylogenetic tree indicated that TelMV from patchouli was a close relative of TelMV-Hanoi (Figure 4), as evidenced by a 1000 bootstrap value.

### 3.5. The P1 Proteins of TelMV from Patchouli and TelMV-Pasfru Are Very Different

**Figure 5:**
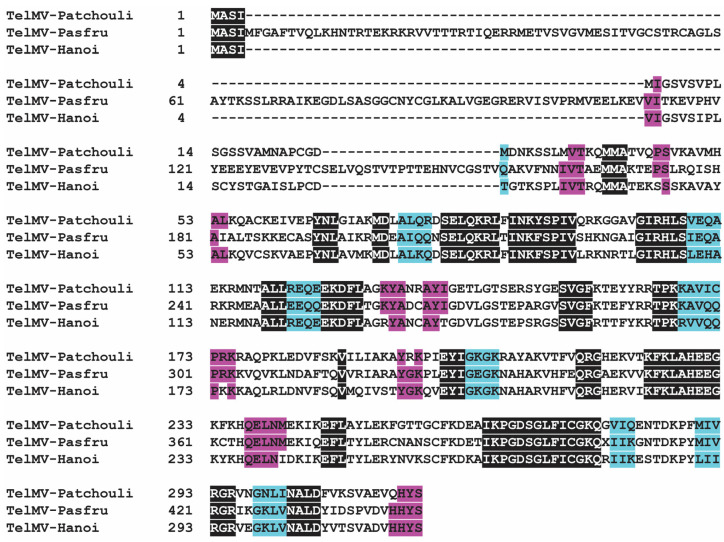
Multiple alignments of P1 amino acid sequence of TelMV patchouli, Pasfru, and Hanoi isolates. The sequence of Pasfru and Hanoi were retrieved from NCBI under accession no. (MG944249 and NC_009742), respectively. The P1 protein of TelMV patchouli is very different from that of TelMV-Pasfru but is similar to that of the Hanoi isolate.

The P1 protein in the genus *potyvirus*, which plays a crucial role in robust infection and host adaptation, is the most variable in both length and amino acid sequence identity among TelMV proteins [33]. To compare the P1, the amino acid sequences of TelMV patchouli, Pasfru, and Hanoi isolates were aligned. The ORF of the TelMV from patchouli is 9248 nt long, encoding 3083 amino acids, whereas the ORF of the TelMV-Pasfru isolate is longer at 9522 nt, encoding 3174 amino acids. Most of the mature viral proteins encoded by these two isolates were similar in length, while the P1 cistron shows considerable variation (Figure 5). The P1 protein of the TelMV from patchouli consists of 317 amino acids; in contrast, the P1 protein of the TelMV-Pasfru isolate contains 408 amino acids, primarily due to differences in the N-terminal region. The N-terminal region of the TelMV from patchouli P1 protein exhibits similarity with the Hanoi isolate. The C-terminal region of P1, which has the serine protease domain, is relatively conserved across all TelMV isolates. Thus, the sequence differences between the TelMV from patchouli and TelMV-Pasf ru isolates are mainly due to the variations in the N-terminal region of the P1 protein.

### 3.6. Biological Testing of TelMV on N. benthamiana Plants

**Figure 6:**
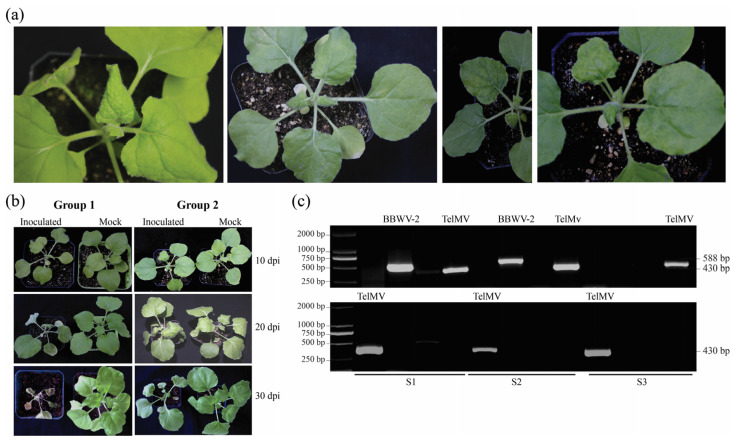
(**a**) Inoculated *N. benthamiana* plants show different symptoms. (**b**) Plants were divided into two groups based on symptoms: group 1 and group 2. Group 1 plants showed severe leaf curling at 10 dpi and, eventually, the death of the whole plant at 30 dpi, whereas group 2 plants showed mosaic patterns and mild leaf curling. (**c**) Confirmation of viruses by RT-PCR from three plants from each group. The presence of TelMV and BBWV-2 in two plants from group 1 was confirmed by the agarose gel electrophoresis of PCR, while TelMV was detected in group 2 plants.

Forty *N. benthamiana* plants were grown under controlled conditions to assess the pathogenic potential of TelMV on the indicator plant. Sap extracted from a virus-infected patchouli plant was used to inoculate the plants mechanically. At 10 days post-inoculation, two distinct symptoms were observed in the inoculated plants (Figure 6a). Based on symptoms, plants were divided into two groups (group 1 and group 2) (Figure 6b). Three plants from each group were selected for RT-PCR to confirm the virus presence using the primer designed based on the patchouli plant RNA-seq data. The RT-PCR assay confirmed the presence of two viruses, TelMV and BBWV-2, in two plants from group 1, while only TelMV was detected in the third plant of group 1. Contrarily, group 2 plants were found to be infected only with TelMV (Figure 6c). These results demonstrate a strong correlation between TelMV and the observed symptoms in *N. benthamiana*, suggesting that TelMV plays a significant role in disease symptom development.

### 3.7. De-Novo Genome Assembly of EAPV from Patchouli Transcriptome

**Figure 7:**
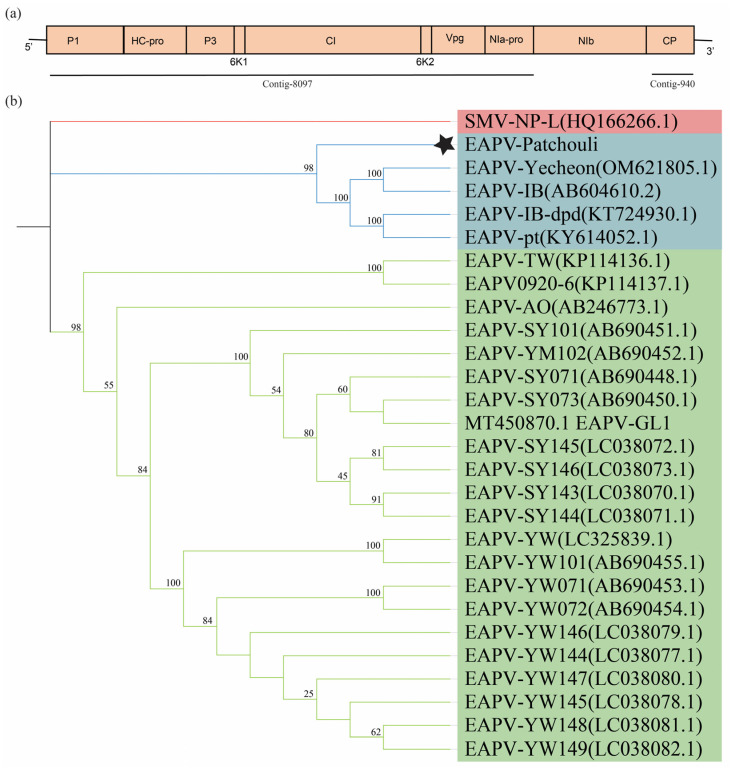
Phylogenetic analysis of East Asian Passiflora Virus (EAPV) from patchouli plant RNA-seq data. The tree was constructed using the neighbor-joining algorithm, and the sequences were aligned in ClustalW using MEGA11. The *p*-distance was employed to calculate the evolutionary relation, and the numbers at the end of the branches represent the bootstrap values derived from 1000 replicates. (**a**) Genomic organization of EAPV reference genome and alignment of contigs with the reference genome. (**b**) Phylogenetic analysis of EAPV. Full names of viruses used in the phylogenetic tree (EAPV = East Asian Passiflora Virus and SMV = soybean mosaic virus). Asterisk denotes the EAPV genome from patchouli.

Numerous attempts were made to identify and amplify the genome sequence of EAPV from infected patchouli plants; however, these efforts were unsuccessful. Instead, the EAPV genome was analyzed using RNA-seq data. A total of 183,896 (0.87%) reads were mapped with the EAPV genome. In Seqman Pro 7.1.0 (Lasergene, GATC Biotech), all EAPV-related contigs were assembled into eight contigs, spanning from 430 nt to 8097 nt (Text S1). Two assembled sequences, spanning 8097 nt and 940 nt, were chosen for further analysis. These sequences cover distinct regions of the genome (Figure 7a). The longest contig, which spans 8097 nt and covers more than half of the EAPV genome, was subjected to comparative analysis using BLAST. The BLASTx and BLASTn comparison revealed that it shares 84.54% aa identity with EAPV-AUT36433 and 77.84% nucleotide identity with 98% query coverage with the complete genome of EAPV, strain IB (AB604610.2).

A further analysis of the other contig (940 nt), comprising the CP of EAPV from the patchouli plant, showed 82.92% to 94.54% aa identity with the CP of other EAPV isolates, with a query coverage range of 75% to 88%. These findings suggest that the EAPV detected from the patchouli plant is a variant of EAPV. The assembled nucleotide sequence of EAPV from patchouli was used to construct the phylogenetic tree. The Ibusuki (IB) and Amami O-shima (AO) are the two physiologically and serologically distinct strains of EAPV from which the ingroups were selected based on their closely linked genomes, while the outgroup was selected from potyviruses. The phylogenetic analysis revealed that the EAPV from patchouli clustered within the same clade as other EAPV-IB isolates (Figure 7b). To our knowledge, this is the first detection of EAPV from patchouli plants. However, further studies are necessary to recover the complete genome and molecular characterization of EAPV from patchouli.

## 4. Discussion

In this study, NCBI RNA-seq data were used to identify the virome of the patchouli plant. An accurate and sensitive diagnosis of viral pathogens is essential for strategically managing viruses. Previously used conventional viral pathogen detection techniques, electron microscopy, enzyme-linked immunosorbent assay (ELISA), nucleic acid hybridization PCR, and indicator host [34] have limitations, as prior knowledge related to sequence or antibody is needed [35]. On the contrary, high-throughput sequencing analysis can identify pathogens without any prior knowledge of the disease [36,37,38,39]. The development of sequencing technologies provides enormous quantities of DNA as well as RNA sequencing data [40,41]. These data can help study novel and known viruses [42]. The NCBI (National Center for Biotechnology Information) is a valuable resource for discovering pathogens, especially viral sequences [43].

The RNA-seq data analysis from NCBI revealed the virome of patchouli plants in China, identifying six viruses: TelMV, BBWV-2, EAPV, PCaCA, PogACRV1_Pog, and PogACRV2_Pog. Previous studies have reported some viruses in patchouli in other countries; for example, PCaCA was detected by HTS in Brazil [44], while ELISA and RT-PCR in Indonesia identified TelMV and Fabavirus [28]. A potyvirus, BBWV-2, and PsTV were identified in India via RT-PCR [45]. Our study identified the viruses in patchouli using an integrated approach that combined RNA-seq data analysis, RT-PCR, and Sanger sequencing. RT-PCR primers were designed explicitly from RNA-seq contigs to target viral genomic regions, confirming the presence of three viruses. This significantly expands the knowledge about patchouli virome in China. However, RT-PCR failures and false negatives can occur due to viral genetic diversity [46]. Low viral titers or uneven distribution in plant tissues may reduce detection accuracy [47].

The host range of TelMV has been steadily extending to include a variety of plants, such as tobacco, patchouli, pigweed, kidney beans, quinoa, Emperor’s candlesticks, and pigweed [48]. In this study, the TelMV genome from patchouli was determined, which was similar in genomic size to a previously reported TelMV isolate in a *Telosma cordata* plant in Vietnam. A comparative analysis of the whole genome sequence of TelMV using BLASTn searches on NCBI databases showed that the TelMV from patchouli showed high nucleotide identity with TelMV Hanoi isolates, indicating a closer relationship. A pairwise comparison of all genome segments sequences indicated less similarity with passionfruit isolates and higher identity with the Hanoi isolate. The genomic sequence of the whole polyprotein and individual genes of TelMV isolates showed higher levels of nucleotide and amino acid sequence similarities, suggesting that several nucleotide substitutions are silent.

P1, the most variable potyviral protein, is classified as type A or B based on its phylogeny, functionality, and chemical properties. P1 has been a subject of scientific interest due to its unclear role in potyviral infections. The N-terminal regions of the P1 protein were subjected to many recombination events among both intra- and intergenic viruses. Our comparison of TelMV isolates from different hosts (passionfruit, cordata, and patchouli) revealed high sequence similarity in the polyprotein from HC-Pro to CP regions, but the P1 region showed lower amino acid identity relative to nucleotide sequences, indicating a higher mutation rate. These findings align with previous research, suggesting that P1 is more variable than other potyviral proteins [49] and may play a key role in host adaptation and pathogenesis [50].

Standard plant virology uses indicator plants to confirm viral presence and assess their pathogenicity. This study mechanically inoculated *N. benthamiana* plants with patchouli leaf sap to test TelMV pathogenicity. TelMV presence in all symptomatic plants was confirmed using RT-PCR assays based on primers derived from the patchouli plant RNA-seq data. These findings highlight a strong correlation between TelMV infection and symptom development in *N. benthamiana*.

EAPV is from the genus *Potyvirus* and consists of filamentous particles of length 680–900 nm and 12–15 nm in width. The virion consists of 10 kb single-strand RNA, which encodes a single polyprotein that cleaves into 11 proteins with different cell functions [19]. The discovery of the EAPV in the patchouli plant greatly expands our understanding of its host range and ecological adaptability. Previously, it was identified in the *Passifloraceae* family. EAPV was primarily found in China, Japan [51], and Malaysia [52], where aphids transmit it non-persistently. The discovery of EAPV in patchouli, for the first time detected outside of its typical host family, raises concerns about the virus’s ability to adapt to new hosts and spread across species. The assembled genome of EAPV from patchouli showed the highest similarity with the EAPV IB strains. The phylogenetic analysis based on the assembled genome sequence grouped the EAPV from patchouli with the IB strain, indicating that it is the variant of this strain. The IB strain has a different host range from the AO strain in *Passifloraceae* [51]. These findings also suggest that the IB strain may be more prone to infecting non-Passifloraceae species. The detection of EAPV in patchouli highlights the potential for the virus to spread to new hosts, emphasizing the need for further research into its complete genome and the mechanisms driving this host shift.

## 5. Conclusions

This study investigated viral infections in patchouli plants, an important medicinal herb and cash crop in China. Six viruses were identified from patchouli transcriptome data, including the first-ever detection of EAPV in patchouli. RT-PCR confirmed the presence of three viruses—TelMV, BBWV-2, and PogACRV1_Pog—in diseased plants. TelMV, the primary focus due to its severe mosaic symptoms, was fully sequenced, revealing a 9691 nt RNA genome and 81.01% identity with the Hanoi isolate. TelMV infectivity was demonstrated in *N. benthamiana* through friction inoculation. While the complete genome of EAPV could not be obtained, a partial genome was reconstructed, confirming the virus with 82.92% to 94.54% CP identity to other EAPV isolates. This study provides the first complete genome sequence of TelMV and the first detection of EAPV in patchouli.

## Figures and Tables

**Table 1:** Primers used to amplify the TelMV whole genome from patchouli.

Fragment	Sequence	Ta for PCR	Length
TelMV 1	F = TGGCATCAATCATGATTGGGTCTGT R = CACCACACTGCTCATTGTCGAAGTC	58	1040
TelMV 2	F = TCAGCCACACCAGAGTTACAATTTTTC R = TATCCATGTTGCTGTGTCACTCATGATGAT	59	863
TelMV 3	F = GATCGTGCGAGATTGCACATGCAAGG R = GCACGCACTCACAAAGGTAC	58	1201
TelMV 4	F = TGGTCCGATTTAAGCTTGTGG R = CAGTTATGTTGCTGGAACCAAA	54	1151
TelMV 5	F = GACCATTAGCTGAAAATGTGAGC R = GGATGCATGCTCCCATCATA	54	962
TelMV 6	F = CCTCCCAGTCACAACACAGAGTGTCACAAC R = GCTTCACCAAAAGTGTGTTCCA	56	973
TelMV 7	F = GATTGGTGGTGGATGGATGATGTGGG R = GCTTCACCAAAAGTGTGTTCCA	58	988
TelMV 8	F = TCATTGAAGGTAAGGATGC R = CAGAACCACTGGCTTATTGTA	50	728
TelMV 9	F = TGTATCAAGTCCCAGAGGCAGC R = CCCATTCTAGAATTGAGACA	50	1120
TelMV 10	F = GACAAACTTGGTTCGTCTTTCGCAGAGCT R = AACATGGTGGTATAACCACTCTG	54	1352
5 RACE-1R 5 RACE-2R 5 RACE-3R 3 RACE-1F 3 RACE-2F	GGTTCCACAATCTCTTTACATGC TACTGATGGCTGCACAGTAGCC GCCACATGGCGCGTTCATTGCAACAC CTGTCACACAGATGAAGGCAGC CTGAGAGGCACACTGCTAGAGATG		

Note: Ta (Annealing temperature) used for PCR reaction.

**Table 2:** No. of contigs and their length related to each virus genome from the total RNA-seq dataset.

Closely Related Virus	Family	No. of Contigs	Length of Contigs	% aa Identity	Query Coverage
Pogostemom alphacytorhabdovirus 1_Pog	*Rhabdoviridae*	10	319–10,344	100	27–99
Pogostemom alphacytorhabdovirus 2_Pog	*Rhabdoviridae*	23	319–10,029	56–100	42–100
Telosma mosaic virus	*Potyviridae*	119	200–1008	45.83–100	44–100
East Asian Passiflora Virus	*Potyviridae*	114	200–3568	50–100	50–100
Patchouli chlorosis-associated cytorhabdovirus	*Rhabdoviridae*	6	642–8865	71.55–90	34–81
Broad bean wilt virus 2	*Secoviridae*	26	200–5776	68.09–100	48–100

Note: Percent amino acid identity and query coverage are related to the best match in BLASTx analysis.

**Table 3:** Comparison of TelMV genome sequence from patchouli plant with Pasfru and Hanoi.

Segment	Genome Position	Length (nt)	Protein Size (aa)	Sequence Identity (nt)	
				Pasfru	Hanoi
5′ UTR	1–189	189	-	81.08	-
P1	190–1140	951	317	69.18	70.59
Hc-Pro	1141–2511	1371	457	74.25	81.04
P3	2512–3552	1041	347	72.41	77.15
PIPO	2965–3184	219	74	80.37	87.21
6K1	3553–3708	156	52	80.67	80.00
CI	3709–5610	1902	634	79.91	83.12
6K2	5611–5769	159	53	-	79.61
NIa-VPg	5770–6339	570	190	75.81	79.32
NIa-pro	6340–7068	729	243	79.78	85.60
Nib	7069–8619	1551	517	81.56	85.37
CP	8620–9438	819	273	87.44	88.48
3 UTR	9439–9691	253	-	88.67	93.36

Note: “-” for not available.

## Data Availability

The data will be made available on request.

## Supplementary Materials

The following supporting information can be downloaded at: https://www.mdpi.com/article/10.3390/v16121837/s1. Text S1. East Asian Passiflora virus (EAPV) genome sequence assembled from RNA-Seq data. Table S1. Primers used for virus identification from infected plants. Table S2. Primers used for virus identification from inoculated *N. benthamiana* plants. Table S3. Blast X analysis of viral contigs from each sample.
