# Supplementary material for: Identification and Molecular Characterization of Telosma Mosaic Virus (TelMV) and East Asian Passiflora Virus (EAPV) from Patchouli in China
Source: Viruses. 2024 Nov 27;16(12):1837. doi: 10.3390/v16121837 (PMC11680427; doi:10.3390/v16121837)
Supplement: Supplementary file 1 [file viruses-16-01837-s001.zip › viruses-3295992-Supplementary.pdf]

Supplementary Text and Tables

Text S1. East Asian Passiflora virus (EAPV) genome sequence assembled from RNA-Seq data.

>EAPV sequence from patchouli plant RNA-SEQ data(contig 8097)

CATAGAAACCAACGTCATCATATTTCCAAGCTTTCTTGAATTAGCACTCAGACTTGCAAGCAAGATTTACAA
ATCTACATTTGACTTCTTTAGCACTATACAACTGATATTCTACTTTGTCTGTACTTTCATTTCTTCGACACACAC
TTATCCACTTCTTCAGATGGCTGCTATCATCTTTGGCAGTTTCTCCGCTCCTATCACCAAAACCACAACTGTGG
TCAAGGCAAAGCGCTTGGTGCCTAGTACCAAGTTAGTCACAAAGAGACTGGTACAAACTGTACCAGTTTCA
GTAATGAAGGATGTGTATGTTGGATGTTCAAGCAGATGCGCGGGTCTAGGCGCATACACAAAGACTTCCCTT
CGGCGCGCTATCAAGGAAGGTGATCTGACAAGTTCTGGGGCTTGTCACAATTGTGGGTTGCAAGGCCTTGTT
GGGGAAGGTCGGAAAAGGGTAGTATCTGTTCCTTTTGTTGAGTATCAAGAAAAGGAGGTGCTTCACACAGA
AGAAGTACCTATAATGATCGAGGAGGAATACGACCTGGAAGTTCCCACCTTTGTCATACCACCACCGGTTGC
TGAGAAGGTAGAGGATTGCAAATTATTGAGTGGAGAGGTCGCCCACTCAGTACCAAAGGCTGAGATGATGG
CCACTGAAAAGCCATCAATTAAACAGATCTCTGGAAAGCTGAGGAGACAAAGCATAAACATGCTTGCTGAG
CACGATAAGAAAATTGCTGCGTTTGATAGGTGTTTACAGCAAAATTCTGAGTTGCAGAAGCGTCTGTTCATTA
ATAAATACAGTCCTATTCAGCAGAAGAGGGGTGGAGTTACCTGTATACGCCACTTAACAAGTGCTCAAGCG
GGAAATCGCAAACTTAGGCTTGAGGAGCTTGCGCAACAGGAAGCTGACTTCCATGCTGGAAAATACTTTGA
GACGTGTGCTGGGCATGTTCAACTACCGACCGCGCCTAGTGCAGGTGCAAGCATCGGATGGAGGACCGCTT
ACTACAAGCGGTCAATTAAGCAGTGCGTACTGAAACGCGCTCAGAAGATTCGTGTCAAAGACAATGGTGCG
CTAGACAAGGTTCTACGTCTAGCAAAAACAATCTCATTGCCTATTGAGTTCATAGGCAACGGCAAACAGCGA
GTGATTAGAGCTCGTTTTGTTCGGAGGAATACTGATATTCTTCCACAAGTACACTTACCACATGCCGATGGGG
TGTATAAAAATCAGGAACTGAACTTGGTTTACGCTGGAAAAATACTTGCTGCACTGTGTAAGTATACTAAAC
CAAGTAGGATTTCAAGTACCACCATTAAACGCGGTGACAGTGGCTTAGTCTTTGATAGACGTTCTGAGGTCA
CGCGCGAAAGCACACAATTACCATATTTCGTTGTTCGCGGACGTCAAGGTGGTAAGTTAGTGAATGCGCTCG
ACTATATAAAATATAAAGAGTATGTTCAACATTACTCACAGGCACCAGAAGCGCAATTCTATGCTGGTTGGA
AAAAGGTGTTTGACAGAATGACACCTCAACATGAAGCTCACTTGTGCACAGTTGACTTCAGTAATGAGCAGT
GTGGTGAAATAGCTGCATTCATCAGCCAGACCTTGCTTCCTGTTAAGAAATTGTCTTGTTTATCATGCAGAAA
ACACTTGCAGGATTTGAGCATGGATGAATACAAACACTTTCTAACCACACACATGGTTTGTTGTGATAACATT
TGGGACGACATTCACAAAACTCAAGGGGTTGAATTGGTTAAGAAGTTTGTACAACAAGCTACGGTTGGAAA
TACAAATTTTGAGACCACAATGGAGATTGTGAACTTGACCCAAAATTACACCACAACTCCTATGCTACAGAT
CCAAGACATCAATAGAGCATTGATGAAAGGGTCTTCAGTAACTCAGCAGGAACTTGGGATAGCAACTAAAC
AGTTGTTAGAAATGACAAGATGGTGGAAGAATCACATGAGTCTTACGGATGAAGATACACTCAAAGTGTTC
AGAAACAAAAGATCATCTAAGGCAATGATCAACCCTAGTCTCCTTTGTGACAATCAGCTGGATCGTAATGGG
AATTTTGTTTGGGGAGAAAGAGGAAGACATTCTAAGAGATTCTTCTCGAACTTCTTTGATGAAATCATCCCAT
CTGAGGGTTACTCAAAATATGTGATTCGACAAAATCCAAACGGGCAAAGAAAGTTGGCAATTGGATCATTA
ATTGTCCCGTTGGATTTTAATCGAGCTAGGCAAGCCTTGCAAGGGGAGAGTATAGCAAAGGAACCACTCAC
GCTAGCTTGCATCTCAAGGCAAAGTGGGAACTTCGTATACCCATGTTGTTGTGTCACACATGATGATGGCAG
ACCATTTTACTCGGAACTCAAGAGCCCAACAAAAAGACACTTGGTGATTGGATCGACGGGGGATCCAAAAT
ACATTGACCTTCCATCGGAGGACACTGATCGCATGTATATAGCAAAGGAGGGATATTGTTATTTGAACATAT
TCTTGGCCATGCTTGTGAATGTCAACGAACAAGATGCAAAAGACTTCACAAAAATGGTTAGAGATGTAATCG
TCCCAAGACTTGGGAAGTGGCCCTCGATGATGGATGTGGCAACAGCAACGTACATACTTACAGTCTTTCATC
CAGAAACACGCAATGCTGAGCTACCAAGGATCCTTGTTGATCATGCAACGCAAACAATGCATGTAATTGATT
CCTTCGGGTCTTTAACAGTTGGTTATCATGTTTTGAAGGCAGGAACGATAAACCAGCTAATTCAGTTTGCTTC
AAATGATCTGCAAGGAGAGATGAAATTTTATAGAGTTGGGGGTATGGCAGATCAACGCATAAAATGTGAGA
CAGCTCTACTCTCTAGCATATTCAAACCAAGGAGAATGATTCAATTGCTCAATGAAGACCCATACATTCTTCT
TCTCGGCATGGTCTCGCCATCAGTGCTAATCCATATGTACCGCATGAGACATTTCGAAAAAGGAATTCAGAC
ATGGATTAGCAAAGAGCAGAGCGTGGCAAAAATTTTTATTATATTGGAGCAATTAACAAAGAAAGTTGTTTT
GAACAATGTGCTCCTTGATCAATTGCAAGCCATCTCTTCTTCCTCAGGACATCTAGTGGAAGTGTTATCAGAT
TGTCCACATCATTCACATTCATACAAGCCAGCTATGAATATGCTTACACAGTTTTTAGAGCGTGACTTGTCCA
ACAAGCAGCTTAGCGAGAATGGTTTTGTAGATCTGCATGAAAACCTTTACGTGGAGGTCGAAAAAATCTATG
TGGAAAGATTAAGTCAGGAATGGCACGCACTAAGCTTGTTGGAAAAATCTTCTGTAACTTGGCAATTGAAAA

AGTTTTCTCATTTTACGGAAAAATCTTTGACAAAGAAAGTTGCAGAAGGAAAAGAAGGATTTTCAAAATCCT
TTGTGAGTGCGTGCTTCATGACTGCACAATCACACCTAAAAAGCGCTCGCACATCATTCTTGAGGAGGTGTG
AAAATGCATATTCTTCTTTAGTTAGGAAGTGTATGAACATACTACTTAAGACTGTGTATCGTTGTTATAGTGA
TATTTTGTATTTGGTTAATTTGTGTATAGTTTTCTCTCTTTTAGTGCAGATGGCAGCTACATTGCATAATGTAGT
GAAAAAGATACAAGTTGACAAGGCAATTTTGCAGCGAATGAAACAAGGGGAAGAAGAGACAACACTTTTG
CATATATATGATTTCTTCTCTAAAGCAGAGGGAGGAACTCCAACAATTGAAGCATTCACAGAACATGTGAAA
GAGGTTCGACCGGATTTGCTGCAAACGCTCAAAAGTTTGACAAATCAACAGGAGAGCGTATCCTGTCAAGC
AAAAACGTCAACTCAGTGTCAGTTTGAGAAAATAGTAGCTTTTATGGCATTGCTTACGATGTGTATTGATAAT
GAGAGAAGTGATGCTATATTCAAAATCCTGAGCAAACTCAAAGTAGTCTTTTCTACAATGGGTGAGGATGTC
AAAGTACAAAGTCTTGATGAAATTGTGAATATTGAGGAGGATAAGAAGCTTACTATTGATTTTGATCTTGAG
ACAAACAAGGAGCCCTCTTCTGTTTCATTTGATGTAAAGTTCGAGGATTGGTGGAACAGGCAGCTACAGCAG
AATAGAGTAATTCCACATTACAGGACGACTGGTGAATTTTTGGAATTCACTAGAGAAACAGCTGCAAAGGT
GGCAAACCAAATTTCCACTTCAACCTCTCCTGAGTTCTTAGTACGTGGAGCAGTTGGATCAGGAAAGTCAAC
AGGTTTACCGCATCATTTATCCAAGAAAGGGAGAGTTCTTCTTCTTGAACCAACTAGACCACTTGCTGAAAA
TGTCAGCAAGCAACTGAGTGGTGATCCTTTCTTCCAAATGGTAACATTGCGAATGAGAGGGTTGAGTAGGTT
TGGTTCAAGTAACATCACAGTGATGACTAGTGGTTTTGCCTTCCACTACTACGTAAATAACCCCACTCAATTG
TCTGAATTTGATTTTATAATAATAGATGAGTGTCATGTCTTAGATAGTTCTACTATAGCATTTAATTGTGCTCTT
AAGGAGTTTGAATTTTCAGGTAAGCTAATAAAAGTTTCAGCCACACCTTCCGGCAGAGAGTGCGAATTCACA
ACACAGCACCCAGTTCAGTTGAAAGTTGAAGATCAGATCTCATTTCAACACTTTGTTAATGCACAGGGAACG
GGATCTAATGCTGATATGGTTCAACATGGCCACAATTTGTTGGTGTATGTGGCTAGCTACAATGAAGTAGATC
AACTGGCACGTCTACTAGTAGAGCGTCAGTACAAAGTTACTAAAGTTGATGGGAGGACGATGCAAAAAGGC
AATGTAGAAATAGTCACATCAGGTTCAGAGGGTAAGCCGCACTTTATAGTTGCAACGAATATCATCGAAAAT
GGAGTGACTATAGATGTGGATTGCGTGATTGACTTTGGTCAGAAAGTTGTAGCAATGTTGGATAGTGACTGC
CGATGCTTAAGATATAATAAGAAACCAGTCACATATGGAGAACGAATTCAGAGGCTTGGTCGAGTTGGGAG
GTGCAAACCTGGTTTTGCACTGAGAATAGGACATACTGAGAAAGGAATTGAGGAAATTCCTGAATACATTG
CCACTGAAGCAGCTTTTCTTTCATTCGCGTACGGATTGCCTGTTACCACGCAAAGTGTGACAACGAATATGCT
TTCAAAATGCACAGTTAAACAGGCACGTAATGCATTGAATTTCGAGCTAACACCATTCTTTGTTATACACTTC
ATTAAGTACGATGGCAGTATGCATCCTGAGATTCACAATCTTCTCAAGTCCTTCAAACTTAGAGAATCAGAG
ATGTTACTTAATAAGCTCGCCATTCCCTTTCAGTATATCAACCAGTGGCTCACAGTAAGAGAATACGATAGA
CAAGGTGTTCATCTGAATTGTTCTGAAAGTACGCGCATCCCATTTTATGCGCATGGAATACCTGATAAGCTTT
TTGAGACGCTGTGGGACACGATTTGCAGATACAAGAGTGACGCTGGTTTTGGGAGACTTTCAAGCGCTAGCG
CAGCTAAAGTTAGCTACACGCTTAGCACAGATCCCAGCGCCGTACCTAGAACTGTCGCCATCATTGATCATT
TATTAGCTGAGGAGATGATGAAAAAGAATCATTTCGACACTATTGGGTCTTCAGTTACGGGGTATTCTTTTTC
ACTTGCTGGGATAGCTGAAGGATTTCGCAAAAGGTATATGAAAGACTATACTCAGCACAATATTGCAATTCT
TCAACAGGCAAGGGCGCAGTTGCTAGAGTTTGACAGCACAAGAGTTGACATAAATAACTTACACGGAATGG
AAGGGGTTGGTGTTCTCAATGCAGTGCAATTGCAGAGTAAGCATGAAGTGTGTAAGTTCCTGAATTTGAAAG
GTAAATGGGATGGTAAGAAGTTCATGAATGATGCTGTGGTGGTGATATTTGCTCTCATTGGCGGGGGTTGGA
TGATGTGGGAATATTTCACCAAGAAAATGAAGGAATCAGTGGTCACTCAAGGGAGAAAGCGCACCCTTCAA
AAGCTCAAGTTCAGGGATGCCTATGATCGAAAGATTGGTCGGGAAGTATATGCGGATGATAACACAATGGA
GCACACATTTGGTGAAGCATATACGAAGCGTGGGAAAAAGAAAGGTAGTACTGAGACAAGAGGAATGGGG
CGGAAAACCAGAAACTTCATCCACATGTATGGGATTGAACCAGAAAATTATAGCATGATTCGATTTGTAGAT
CCCATAACAGGCCACACGATGGATGAAAACCCTAGAGTCGACATAAGGCTTGTGCAAGAAGAATTTGGTGA
AATACGCAAAAGAATGCTTGAAGAAGAAGCAATTGACAAACAGCACATTATCCATAATCCTGGATTGCAAG
CATACTTCTTCGGGAAAAACACAGAGGATGTATTAAAAGTTGATTTAACACCGCACAAGCCAACACTTCTCT
GTGCGAATTCAAATACAATCGCAGGTTTCCCAGAAAGGGAAGATGAATTGAGGCAGACTGGATTGCCTCAA
AGGGTATCAAAATCTGAGGTACCCAAAGCAAACGAGTGTGTTGAGCTCGAAAGCAAGTCAGTGTACAAAGG
GCCAAGGGATTACAGTGCCATCGCAACACTGATTTGCCAGTTAACCAATGCATCTGATGGGCATAGAGAAA
CAATTTACGGAATTGGTTATGGCGCCTACATCATCACAAATGGGCACTTATTTAGGCGTAACAATGGAATTCT
AACTGTGAGGACATGGCATGGCGAGTTTGTAATAAATAACACCACACAGTTAAAAATTCATTTTATTGAAGG
TAAGGATGCTATCCTCATTCGAATGCCCAAGGACTTTCCTCCTTTCTCTAAAAGAAGTTTCTTTCGTCAGCCCC
GCAAAGAAGAAAGAGTTTGCATGGTCGGCACAAATTTTCAAGAAAAGAGTCTTAGGGCGACAGTATCTGAA
TCCTCAATAATAGTTCCTGAGGGAGTTGGGTCCTTTTGGATCCACTGGATAACCACACAAGAAGGTTATTGTG
GTCTACCTCTAGTTTCTGTTAATGATGGGTTCATTGTAGGCTTTCATGGCTTGAAGTCAAATGATTCTGAAAAG

AACTTCTTTGTCCCATTCATAGATGAGTTTGAGGATAAATACTTAAAGAATGTGGACACGTTGTCTTGGGATA
AGCACTGGCTCTGGCAGCCTGATAAAGTAGCATGGGGTTCACTCAACTTGGTGGATGAGCAACCTAGAGAG
GAATTCAAAATTTCCAAACTCATCTCTGACTTATTTGGAGACTCGGTAATTGTGCAGAGTAAGAGAGAAAGG
TGGGTTCTGGATGACATGGAAGGGAACTTAGTAGCATGTGGACAAGCTGATAGCGCTCTAGTGACAAAACA
CGTCATAAAAGGCAGATGCCCACATTTTGAGCAGTACCTCGCACAGCATGAAGATGCTGAAAAATTCTTTAG
GCCATTAATGGGAGCTTATCAGCCAAGCAAGCTGAACAGAGAAGCATTCAAGAAGGATTTCTTTAAATATA
ACAAGCCAGTTGTTCTCAATGAAGTCGATTTTACTGCATTTGAGCAGGCAGTGTGTGGGGTGAAGGACATGA
TGATGAGCTTTGGTTTTCATAATTGTACATACATCACTGATCCCGATGAAATTTTCAATTCCCTCAATATGAAG
GCTGCTGTTGGTGCACAGTATAGAGGTAAGAAGAGTGAATACCTGGCTGATATGAATCAGTTTGACCGAGA
AAAGCTTCTATATTTAAGCAGTGAAAGACTGTTTTATGGTAAGAAAGGTCTCTGGAATGGGTCACTTAAAGC
TGAATTGAGACCCAATGAGAAAGTTTTGGCGAACAAGACCAGAACATTCACTGCTGCACCAATAGACACAC
TGCTGGGTGCGAAGGTTTGTGTGGATGATTTTAACAACCAGTTTTACAGCTTGAATTTAATTTGTCCATGGACT
GTTGGAATGACGAAGTTTTATGGTGGATGGGA

>EAPV CP-patchouli (contig 940)

CTATCGATGCTAGAGGCGATGGAGGAAAAAGGGATAGAGGTAAGGACAAACAGGCAAGAACTCTAGCAAA
CCCTGAAAATCCAAATCCCAGTAATCCAAATAATGATAGCAATTCATCAATTGTGCGTGATAAGGACGTCAA
CGCTGGCTCTAAAGGAAAGGTGGTGCCACGACTACAAAAGATCACCAAGAAAATGAATTTGCCCACTGTGA
GAGGCAAAATTATACTTGATCTCAATCACTTGATCGAGTATTCACCAAACCAGGTGGATCTTTTTAATACTAG
GGCAACCAAGGCACAATTTGAATCCTGGTACATGGCAGTTCAGAAGGAATATGAGCTGGATGATAATCAGA
TGAGTGTTATCATGAATGGTTTCATGGTGTGGTGCATTGACAATGGCACTTCACCAAATATCAATGGCATGTG
GGTTATGATGGACGGAGATGAACAGATCGAATACCCACTAAAACCATTGGTTGAAAATGCCCAGCCTACTTT
GAGACAAATAATGCATCACTTTTCCGATGCGGCTGAGGCATATATAGAAATGAGAAATTCCAAAGAACCGT
ACATGCCTAGGTATGGCACTCTTAGGAATTTGAGGGACTTGAGTTTGGCTCGTTATGCTTTTGACTTCTATGAA
GTTACATCTAAAACACCAAACAGAGCAAGAGAAGCGGTAGCTCAAATGAAGGCCGCTGCCCTCGCGAACG
TTTCCACTAGGTTGTTTGGCCTTGATGGAAATGTTTCAACAAATAGCGAGAATACCGAAAGGCACACTGCTA
GGGATGTTAGTCAAAACATGCACACCCTTTTGGGTGTGAGCTCTATGCAG

Table S1. Primers used for virus identification from infected plants.

Virus	Sequence	Region of genome	Ta for PCR	Amplicon size
TelMV	F=GAGCAACCAAGCAGCAGTTTG<br>R=CAACGAGGGCGGCTGCCTTC	CP	60	432
EAPV	F=GATGACAATCAGATGAGCGTC<br>R=ACCATTCATGATGACGCTCAT<br>F=TGGTGCATTGACAATGGCAC<br>R=GCAGTGTGCCTTTCGGTATTCTCGC	CP	52<br>59	388<br>401
BBWV-2	F=GGATTGGACATGAGCAATTGCTC<br>R=CTACCAGCCCCGTATGGAAAC<br>F=GCGCACCATATATTTCGAAGATC<br>R=ACGAGTGGCATACTCGTACAA	RNA-1	56<br>52	301<br>558
PCaCA	F=GTGGCAGCTTCCCATACACTG<br>R=CAGGGCATGCATGCACTGACTC<br>F=GGCGATATTCTCTCCAGTATCCTTAT<br>R=GCCTGATGAACAATGGAACTTCGACATAGG	P protein	59<br>56	437<br>651
PogACRV2_Pog	F=GCAAAGGGTGATGAAGATAGC<br>R=GCTCCTCTAAATGTGCTGGAGTC<br>F=GCTATGAATGCATACATCCCGTGG<br>R=CAGCAGTAAAACATCCCGAGAC	L	52<br>54	749<br>525
PogACRV1_Pog	F=TGAGAGTGTAGTCGACAACATC<br>R=AACTTCCTCCCTTGGTCATC<br>F=GCTGCCTTTATAATGAAGTTGAACGG<br>R=ATCTCGTGGTCCTTAATCAAGTC	RdRp	52<br>54	1767<br>432

Note: For TelMV, a pair of primers were used in the RT-PCR, whereas two pairs of primers were used for each of the other viruses.

Table S2. Primers used for virus identification from inoculated *N. benthamiana* plants.

Virus	Sequence	Region of genome	Ta for PCR	Amplicon size
TelMV	F=GAGCAACCAAGCAGCAGTTTG<br>R=CAACGAGGGCGGCTGCCTTC	CP	60	430
EAPV	F=TGGTGCATTGACAATGGCAC<br>R=GCAGTGTGCCTTTCGGTATTCTCGC	CP	59	401
BBWV-2	F=GCGCACCATATATTTCGAAGATC<br>R=ACGAGTGGCATACTCGTACAA	RNA-1	52	558
PogACRV1_Pog	F=GCTGCCTTTATAATGAAGTTGAACGG<br>R=ATCTCGTGGTCCTTAATCAAGTC	RdRp	54	432

Note: Ta (annealing temperature) for PCR reaction.

Table S3. Blast X analysis of viral contigs from each sample.

Sample	Closely related virus	Family	No. of contigs	Length of contigs	% Amino Acid identity	% Query coverage
NX-1	Pogostemen alphacyrhabdovirus 2	*Rhabdoviridae*	6	874-10029	86.18-100	44-100
	pogostemom alphacytorhabdovirus 1	*Rhabdoviridae*	5	319-10029	100	33-99
	Telosma mosaic virus	*Potyviridae*	17	202-546	54.79-98.53	88-100
	Broad bean wilt virus 2	*Secoviridae*	6	205-1769	89.16-100	97-100
NX-2	Pogostemen alphacyrhabdovirus 2	*Rhabdoviridae*	10	488-9841	56-99	86.31-100
	pogostemom alphacytorhabdovirus 1	*Rhabdoviridae*	3	1264-10344	100	57-84
	Telosma mosaic virus	*Potyviridae*	19	202-476	45.83-98.41	56-100
	Broad bean wilt virus 2	*Secoviridae*	3	217-5725	93.34-97.81	97-99
NX-3	Pogostemen alphacyrhabdovirus 2	*Rhabdoviridae*	7	319-7476	82-100	42-100
	pogostemom alphacytorhabdovirus 1	*Rhabdoviridae*	2	4875-8395	100	28-73
	Telosma mosaic virus	*Potyviridae*	11	202-330	59.72-96.19	96-99
	Broad bean wilt virus 2	*Secoviridae*	9	200-5776	68.09-100	51-100
PX-1	Patchouli chlorosis-associated cytorhabdovirus	*Rhabdoviridae*	2	3573-8859	82.19-90.12	34-70
	Telosma mosaic virus	*Potyviridae*	22	201-770	59.05-100	82-100
	East Asian Passiflora virus	*Potyviridae*	34	202-2199	56.9-98.11	60-100
	Broad bean wilt virus 2	*Secoviridae*	2	764-1013	93.04-97.64	80-99
PX-2	Patchouli chlorosis-associated cytorhabdovirus	*Rhabdoviridae*	2	642-8865	79.27-82.19	70-76
	Telosma mosaic virus	*Potyviridae*	24	201-1008	55.81-100	44-100
	East Asian Passiflora virus	*Potyviridae*	38	202-2405	50-100	50-100
	Broad bean wilt virus 2	*Secoviridae*	3	408-1039	91.85-98.27	52-99
PX-3	Patchouli chlorosis-associated cytorhabdovirus	*Rhabdoviridae*	2	1703-7261	71.55-84.95	67-81
	Telosma mosaic virus	*Potyviridae*	26	200-869	58.76-100	60-100
	East Asian Passiflora virus	*Potyviridae*	42	200-3568	62.61-100	56-100
	Broad bean wilt virus 2	*Secoviridae*	3	346-663	84.62-98.29	48-99
